# Integrated metabolomic and transcriptomic analyses reveal aroma diversity and its regulatory networks in aromatic acidic citrus

**DOI:** 10.3389/fpls.2026.1785725

**Published:** 2026-02-20

**Authors:** Changjiang Cui, Lifang Sun, Xiu Huang, Zhenpeng Nie, Yihang Zhu, Luoyun Wang, Yi Yang, Xiaodong Xing, Fuzhi Ke

**Affiliations:** 1 Institute of Citrus Research, Zhejiang Academy of Agricultural Sciences, Taizhou, China; 2 National Center for Citrus Variety Improvement, Taizhou, China; 3 Institute of Digital Agriculture, Zhejiang Academy of Agricultural Sciences, Hangzhou, China

**Keywords:** aroma, citrus, metabolomics, transcriptomics, VOCs

## Abstract

Aroma is a key quality indicator of citrus, especially an important sensory trait of aromatic acidic citrus germplasm. Nevertheless, systematic research of aroma formation and its molecular regulation in aromatic acidic citrus remain limited. In this study, eleven representative aromatic acidic citrus varieties, including, Kabosu, Xiang yuan, citrumelo, citrange, and seven yuzu cultivars, were selected to characterize their aroma profiles and underlying regulatory mechanisms. Electronic nose analysis showed that aromas of aromatic acidic citrus were mainly perceived by sensors W5S, W1S, W1W, W2S, and W2W. In addition, the aroma characteristics of Kabosu and citrumelo are distinctly different from other varieties. Volatile metabolomic analysis revealed that terpenoids were the predominant aroma compounds of citrus. Key volatile organic compounds (VOCs) were identified based on relative odor activity values (rOAV > 1), with 1-p-menthen-8-thiol, 3−mercapto−3−methylbutyl formate, and 3−Octen−2−one exhibiting higher rOAV across all varieties, largely contributing to the citrus aroma characteristics. Among key VOCs, safranal showed the greatest inter-varietal variation, with the highest variable importance in projection value, and was identified as one of the characteristic VOCs. Weighted gene co-expression network analysis further identified the green (1-p-menthen-8-thiol), tan (3−mercapto−3−methylbutyl formate and 3−Octen−2−one), and graygreen (safranal) gene modules as being significantly correlated with these key VOCs. Notably, the hub genes *Cs_ont_5g010460*, *Cs_ont_5g018210*, and *Cs_ont_3g018770* within the respective modules were strongly associated with the accumulation patterns of the corresponding VOCs, suggesting their potential regulatory roles in aroma-related metabolic pathways. Together, these results provide an integrated view of aroma formation and its transcriptional regulation in aromatic acidic citrus, offering new insights into citrus aroma biosynthesis and germplasm utilization.

## 1 Introduction

Citrus are among the most widely cultivated and highest-yielding fruit crops worldwide in terms of both production volume and cultivated area. According to FAO, in 2024, the global citrus production area was about 10.6 million hectares and production was about 165.63 million tonnes. Owing to their rich nutritional value (containing pectin, polyphenols, flavonoids, minerals, and vitamin C) and distinctive fragrance, citrus fruits are highly favored by consumers (Chen et al., 2019; Ye et al., 2026). The *Citrus* genus, belonging to the Rutaceae family, comprises a wide range of species and cultivars. In addition to the three ancestral species—mandarin (*Citrus reticulata*), pomelo (*Citrus maxima*), and citron (*Citrus medica*)—the genus includes numerous hybrids and derivative varieties formed through natural or artificial hybridization (Huang et al., 2023). The abundance of citrus germplasm resources and their diverse traits underpin the broad utilization and high economic value of citrus. A group of citrus varieties or cultivars characterized by intense aroma and high acidity, which we define as aromatic acid citrus (referred to as Kousan kankitsu in Japanese classification). Their fruits are unsuitable for direct consumption due to excessive sourness, bitterness, or astringency. Nevertheless, these citrus fruits are cherished for their refreshing and distinctive fragrance (Anwar et al., 2016; Stohs, 2017). For instance, yuzu, Xiang yuan, Kabosu, citrumelo, and citrange are commonly aroma-rich acidic citrus. The yuzu (*Citrus junos*) originated in the upper reaches of the Yangtze River in China and was later introduced to Japan and Korea during the Tang Dynasty. It is currently cultivated mainly in China, Japan, and Korea (Ke et al., 2025); Yuzu fruits emit an intense and distinctive aroma, and their fresh fragrance has led to extensive applications in beverages, condiments, traditional medicine, and health supplements (Miyazato et al., 2013; Matsumoto et al., 2016); Xiang yuan (*Citrus wilsonii*) is a wild citrus, which originated from China. Xiang yuan juice exhibits excessive acidity, yet its fruit aroma is highly appealing—featuring a pronounced lemon undertone blended with floral notes and a passionfruit-like fragrance. This variety is primarily cultivated for ornamental purposes or within the fragrance industry (Demarcq et al., 2021). Kabosu (*Citrus* sp*haerocarpa*) is a one of the most popular sour citrus fruit in Japan, characterized by its attractive and distinctive aroma (Minh Tu et al., 2002). Citrumelo and citrange are hybrids of trifoliate with ‘Duncan’ grapefruit and sweet orange, respectively (Stover et al., 2021), and they have high acidity in the pulp, but have a rich aroma. Clearly, the most distinctive feature of this citrus fruit lies in its unique aroma, which is precisely the source of its utilization and research value.

Aroma is a key determinant of citrus flavor quality and a major factor influencing consumer preference. Citrus notes are a key fragrance profile in the flavor and fragrance industry. Extracts derived from citrus peels are highly valued for their pleasant aromatic properties and are widely used in perfume manufacturing (Demarcq et al., 2021). In addition, citrus notes find extensive application in the production of food, beverages, cosmetics, and related products (Vilela et al., 2019). As consumer tastes evolve, demand for exotic and unique citrus profiles continues to grow. In fact, a new trend is emerging: the adoption of lesser-known citrus notes to create novel sensory experiences and uncover previously unexplored taste sensations. Although citrus aromas share a similar overall profile, different citrus species possess distinct sensory characteristics, including the aforementioned aromatic acid varieties. It is essential to analyze the formation and variation of these citrus aromas and their molecular regulatory mechanisms.

The aroma of citrus fruits is determined by the composition and abundance of volatile organic compounds (VOCs) present in citrus essential oils. VOC profiles vary considerably among citrus species and tissues, with the highest concentrations typically found in fruit peels (Park et al., 2024). To date, more than 200 VOCs have been identified in citrus, including terpenes, ethers, alcohols, ketones, oxides, esters, and aldehydes (Zhang et al., 2017). Among these, terpenoids constitute the most abundant and diverse class of aroma compounds in citrus (Ke et al., 2025). Aromatic substances represent a subset of VOCs that can be perceived by the human olfactory system and are associated with pleasant sensory perceptions. Importantly, not all VOCs contribute to aroma perception, which depends on their relative odor activity values (rOAVs), calculated based on odor detection threshold and compound concentration (Grosch, 2001). Generally, a higher rOAV value indicates a more potent odor perceived by humans and is considered a key VOCfor citrus aromas. The variety of aromatic compounds and their distinct rOAV values—known as characteristic VOCs—confer unique aromatic profiles to each citrus cultivar (Zhou et al., 2020). The odor detection threshold is the lowest concentration of a chemical substance in the air that humans can still detect. It is primarily influenced by the chemical properties of the substance, including molecular structure and polarity (Chiera et al., 2025). For example, monoterpenes such as 1-p-menthene-8-thiol, identified decades ago as a key aroma compound in citrus fruits, exhibit an extremely low odor detection threshold in air (0.000034 ng/L) and significantly contribute to the overall scent of the fruit (Schoenauer and Schieberle, 2016). The concentration of the compound is influenced by the VOC biosynthesis pathway. This process involves the intricate interplay of diverse metabolic pathways and is regulated by multiple molecular factors. Among these, genetic regulation plays a central role, including genes encoding biosynthetic enzymes and transcription factors (Dudareva et al., 2013). For example, the terpene synthase gene *CreTPS3a* is responsible for the synthesis of γ-terpinene, a characteristic citrus aroma compound, and its expression is regulated by the transcription factor CreARF2 (Wen et al., 2025). Currently, many important VOCs biosynthetic regulatory pathways remain unclear. Elucidating the biosynthetic pathways and regulatory networks underlying VOC formation is therefore essential for aroma-related metabolic engineering and molecular breeding in citrus.

Currently, systematic comparative studies on VOC profiles and their molecular regulatory mechanisms in aromatic acid citrus are scarce. We selected representative aromatic acid citrus cultivars, including yuzu (cv. ‘Ziyangxiangcheng’, ‘Xiecheng’, ‘Kitou’, ‘Tada Nishiki’, ‘Zairai’ and ‘Line131’), Kabosu, Xiang yuan, citrumelo and citrange, for integrated analysis. Through volatile metabolomic profiling, we identified key VOCs contributing to citrus aroma formation and characteristic VOCs exhibiting cultivar-specific variations. By integrating transcriptomics data, we analyzed the regulatory networks governing the biosynthesis of key and characteristic VOCs and identified candidate regulatory genes. This study provides theoretical insights into the formation and molecular regulatory mechanisms of aroma quality in aromatic acid citrus, offers a theoretical basis for citrus utilization, and identifies potential targets for future citrus molecular breeding.

## 2 Results

### 2.1 Characterization and genetic analysis of aromatic acid citrus

**Figure 1:**
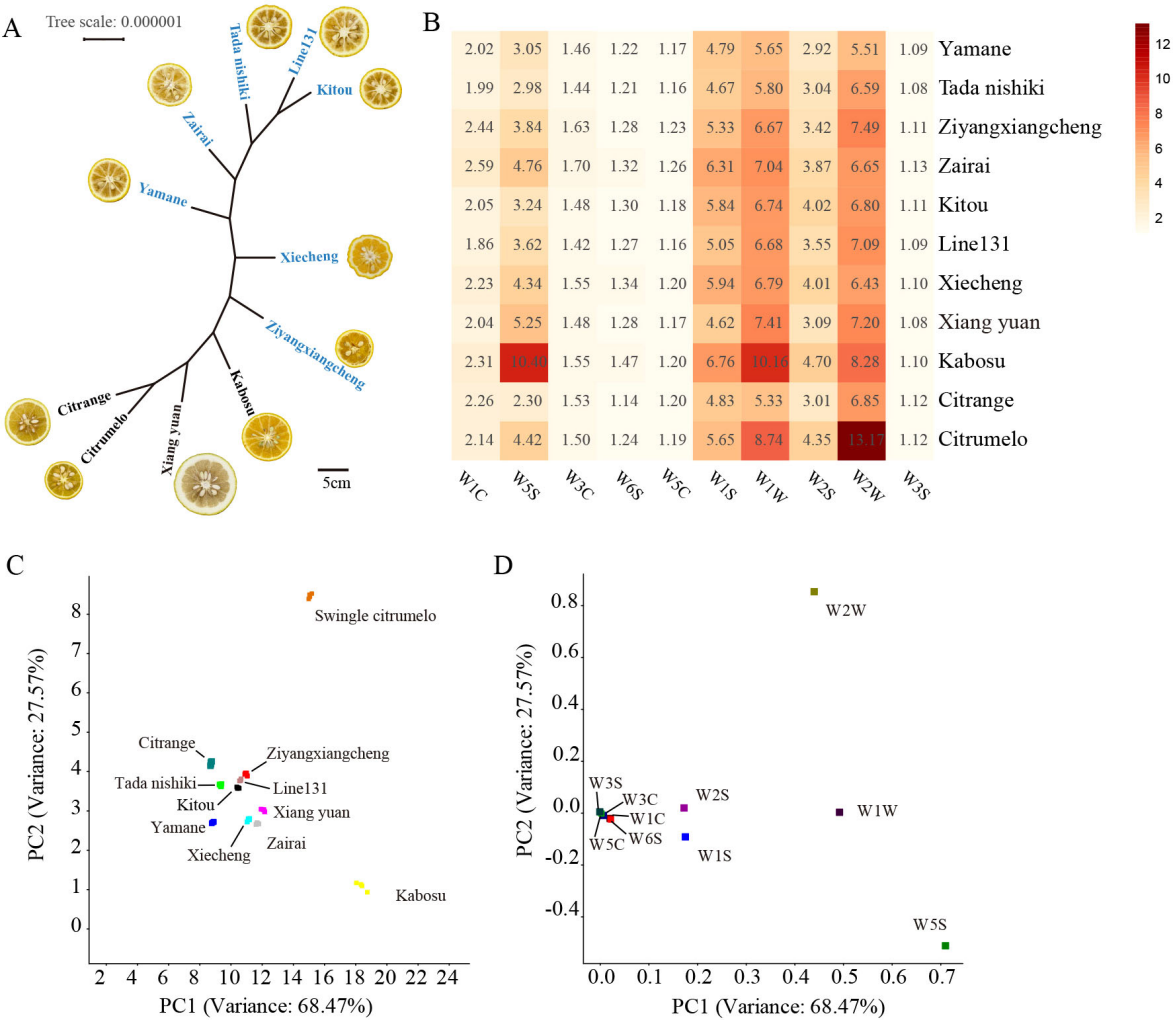
Genetic and trait analysis of different citrus varieties. **(A)** Phylogenetic tree of different citrus varieties. Varieties labelled in blue belong to the yuzu group, with corresponding cross-sections of their fruit shown. **(B)** Heatmap of e-nose analyser sensor response values. **(C)** Principal component analysis of aroma profiles among citrus varieties based on e-nose data. **(D)** Loading analysis (LDA) of E-nose for citrus samples.

To investigate the genetic relationships and phenotypic diversity among these citrus varieties, phylogenetic analysis and morphological observations were performed. Phylogenetic analysis was conducted using the TPM1uf+I+G4 model parameters based on 222,094 genome-wide single-nucleotide polymorphism (SNP) sites. Yuzu (*Citrus junos*) originated in the upper reaches of the Yangtze River in China and is considered a hybrid between Ichang papeda and mangshanyeju mandarin. It was later introduced to Japan and Korea during the Tang Dynasty (Rahman et al., 2001; Wu et al., 2021). In this study, ‘Ziyangxiangcheng’ and ‘Xiecheng’ are indigenous Chinese cultivars; ‘Kitou’, ‘Tada Nishiki’, and ‘Zairai’ are native Japanese cultivars; ‘Line131’ is a regional Korean cultivar. Phylogenetic results indicate that yuzu cultivars exhibit closer genetic relationships among themselves compared to other citrus types. Chinese yuzu cultivars exhibited a more distant genetic relationship with Japanese and Korean yuzu varieties, consistent with their historical dispersal routes (**Figure 1A**). Kabosu (*Citrus* sp*haerocarpa*) originated in China. It is believed that the fruit was introduced to Japan during the Edo period. Legend has it that a Japanese physician purchased the fruit from a monk and subsequently cultivated it in Oita Prefecture. It is speculated to be a hybrid of ichang papeda and bitter orange, but its origin is unknown (Tomiyama et al., 2011). Xiang yuan (*Citrus wilsonii*) is a wild citrus that originated from China. Recent research suggests that Xiang yuan may be a hybrid variety between pummelo and yuzu, with pummelo likely serving as its maternal parent (Demarcq et al., 2021). Kabosu and Xiang yuan are more closely related to Chinese yuzu, possibly due to their shared origin in China and similar parental relatives (**Figure 1A**). Citrumelo and citrange are hybrids of trifoliate with ‘Duncan’ grapefruit and sweet orange, respectively (Stover et al., 2021). They share common trifoliate parents and thus cluster together phylogenetically (**Figure 1A**).

Consistent with these genetic relationships, yuzu cultivars with closer evolutionary proximity displayed greater similarity in fruit morphology, particularly in size and color, whereas substantial phenotypic variation was observed among the other citrus types, such as the thicker albedo found in the peel of the Xiang yuan fruit (**Figure 1A**).

### 2.2 Olfactory comparison of aroma characteristics among citrus varieties

Aroma is a critical quality trait of aromatic acid citrus. To evaluate aroma characteristics among the selected citrus varieties, electronic nose (E-nose) analysis was performed using peel samples from all 11 cultivars. Most citrus varieties exhibited similar aroma response patterns, whereas Kabosu and citrumelo showed distinct aroma profiles (**Figure 1B**). Principal component analysis (PCA) of the E-nose data further confirmed that Kabosu and citrumelo were clearly separated from the other citrus varieties along the principal components, indicating substantial differences in aroma characteristics (**Figure 1C**). Among the ten metal oxide sensors, W5S, W1S, W1W, W2S, and W2W showed the strongest responses to citrus aromas (**Figure 1B**). Consistently, loading analysis demonstrated that these five sensors contributed most strongly to the variation captured by PC1 and PC2 (**Figure 1D**), suggesting that they are key sensors for discriminating aroma differences among aromatic acid varieties.

### 2.3 Comparison of VOCs based on content in different citrus

**Figure 2:**
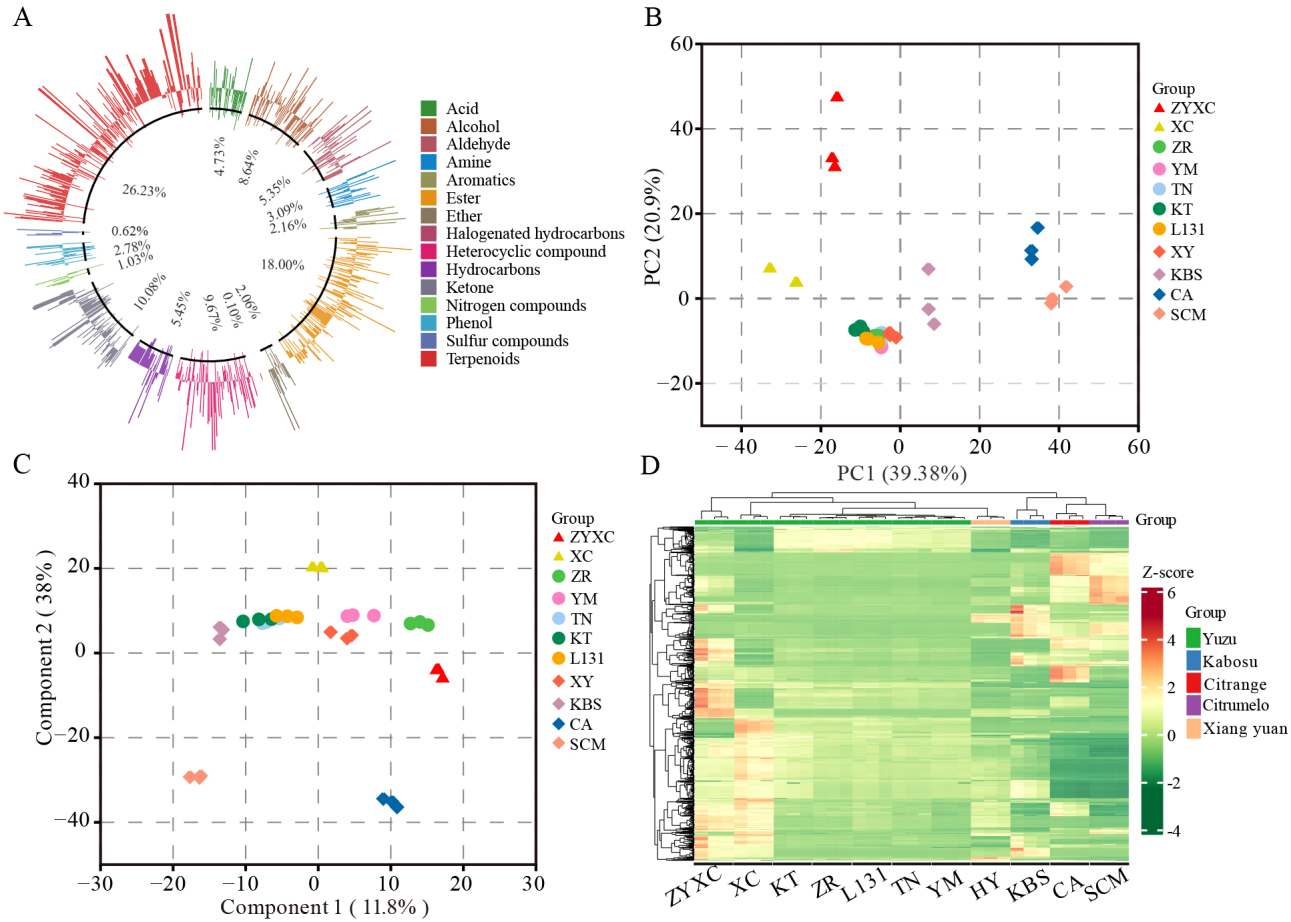
VOCs analysis of different citrus varieties. **(A)** Classification and proportion of VOCs in a Circle Diagram. **(B)** Principal component analysis of aroma compounds among citrus varieties based on VOCs. **(C)** OPLS-DA of VOCs for citrus samples. **(D)** Heatmap of all the VOCs content in different citrus fruits.

To further elucidate the chemical basis of citrus aroma formation, volatile organic compounds (VOCs) were analyzed using headspace solid-phase microextraction coupled with gas chromatography–mass spectrometry (HS-SPME–GC–MS). The results revealed a total of 972 VOCs detected across all citrus samples, comprising 26.23% terpenoid, 18.00% ester, 10.08% ketone, 9.67% heterocyclic compound, 8.64% alcohol, 5.45% hydrocarbons, 5.35% aldehyde, 4.73% acid, 3.09% amine, 2.78% phenol, 2.16% aromatics, 2.06% ether, 1.03% nitrogen compounds, 0.62% sulfur compounds, 0.10% halogenated hydrocarbons. Of these, terpenoids represented the most abundant and diverse class of VOCs (**Figure 2A**; **Supplementary Table S1**). The E-nose sensor responses were consistent with VOC composition. Sensor W1W is primarily sensitive to inorganic sulfides and many terpenes, with its elevated response values correlating with high levels of terpenoid VOCs (**Figures 1B**, **2A**). Similarly, W2S is primarily sensitive to most alcohols, aldehydes, and ketones; W2W, primarily sensitive to aromatics and organic sulfides. The elevated response values of W2S and W2W are consistent with the high abundance of the corresponding VOCs (**Figures 1B**, **2A**).

Multivariate statistical analyses were performed to further evaluate differences in the VOCs profiles. In PCA analysis, all varieties of yuzu exhibited relatively clustered distributions. We observed relative differences between the China-native varieties ‘Ziyangxiangcheng’ (ZYXC), ‘Xiecheng’ (XC) and, other yuzu, including ‘Kitou’ (KT), ‘Tada Nishiki’ (TN), ‘yamane’ (YM), ‘Zairai’ (ZR), and ‘Line131’ (L131) (**Figure 2B**). VOCs of Xiang yuan (XY) and yuzu exhibit similar traits, particularly in relation to KT, TN, YM, ZR, and L131 (**Figure 2B**). Furthermore, the results of orthogonal partial least squares discriminant analysis (OPLS-DA) were consistent with PCA (**Figure 2C**). Heatmap and hierarchical cluster analysis of VOCs across all samples revealed that KT, TN, ZR, and L131 exhibited the most similar VOC profiles. Compared to ZYXC and XC, XY exhibited greater similarity to KT, TN, ZR, and L131. Furthermore, the aromatic compounds of yuzu differed from those of Kabosu (KBS), citrumelo (SCM), and Citrange (CA) (**Figure 2D**).

### 2.4 Identification of key VOCs contributing to citrus aroma

**Figure 3:**
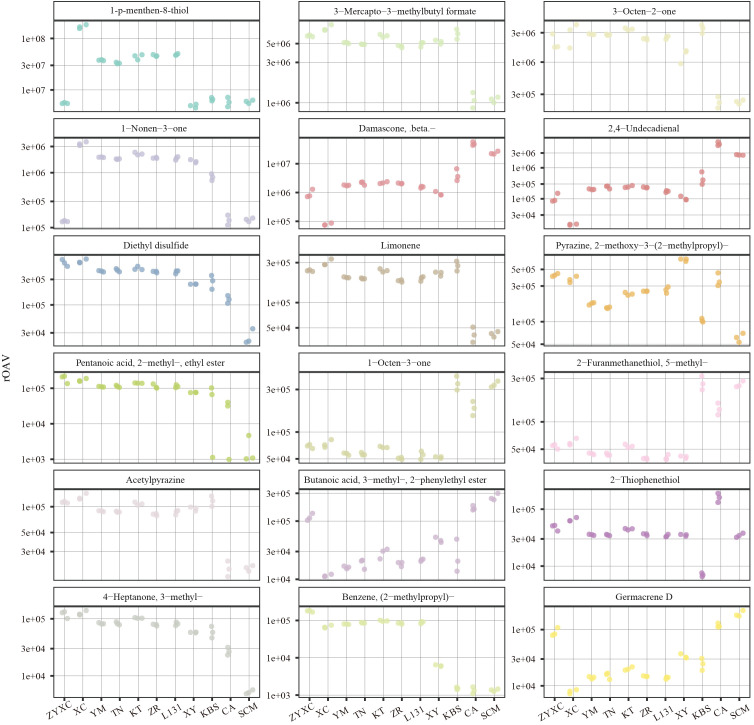
Scatter plot of rOAV values for the top 10 VOCs in each citrus variety.

The sensory impact of VOCs depends on both their concentration and odor threshold, expressed as relative odor activity value (rOAV). VOCs with rOAV > 1 were considered to contribute significantly to citrus aroma. After excluding compounds lacking odor threshold data, 308 key VOCs were identified, consisting of 77 terpenoids, 46 esters, 38 heterocyclic compounds, 33 alcohols, 27 aldehydes, 23 ketones, 21 phenols, 12 aromatics, 8 ethers, 6 acids, 6 hydrocarbons, 4 nitrogen compounds, 4 amines, 3 sulfur compounds (**Supplementary Table S2**). Based on rOAV values, the top 10 VOCs from each citrus variety were selected, yielding 18 VOCs with consistently high aroma contributions (**Figure 3**). These compounds impart diverse sensory notes such as fruity, mushroom-like, sulfurous, green, floral, and sweet notes to citrus fruits (**Supplementary Figure S1**). Among them, 1-p-menthen-8-thiol (rOAV: 4895599−169411622), 3−mercapto−3−methylbutyl formate (rOAV: 1083440−7624089), and 3−octen−2−one (rOAV: 224445−3031934) ranked among the top contributors across all citrus varieties and were particularly enriched in yuzu cultivars (**Figure 3**). These compounds make a major contribution to the formation of citrus aromas. In addition, we found that Damascone, beta- and 2,4-Undecadienal, 1-Octen-3-one, and 2-Furanmethanethiol, 5-methyl- exhibited highly similar accumulation patterns across the different varieties (**Figure 3**).

### 2.5 Characteristic VOCs distinguishing citrus varieties

**Figure 4:**
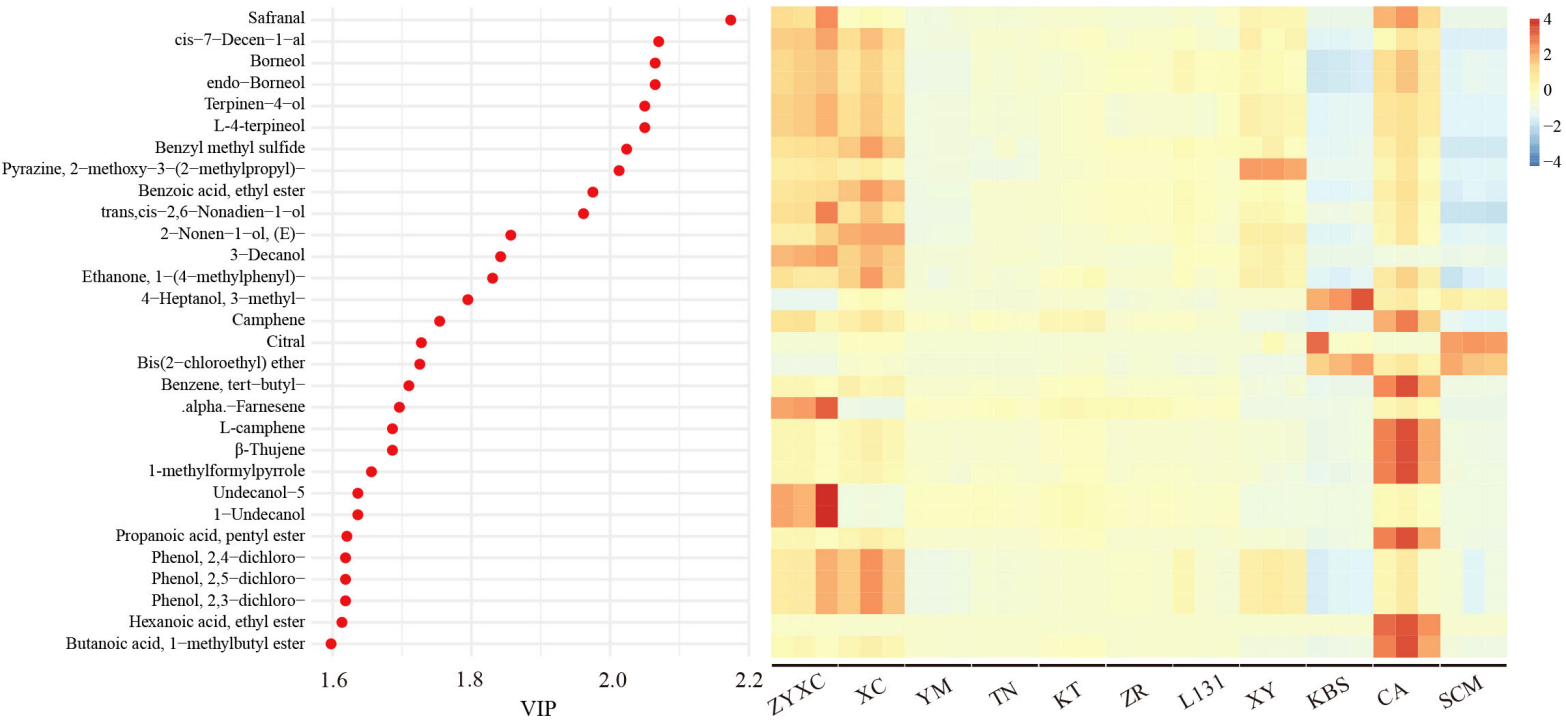
VIP values for characteristic VOCs (left panel) and corresponding rOAV value heatmap (right panel).

To identify characteristic aroma compounds distinguishing citrus varieties, OPLS-DA based on rOAV values of key VOCs was performed. OPLS-DA is a supervised pattern regression analysis method based on orthogonal partial least squares regression. Through interactive residual validation ANOVA, the explanatory variables (R²) and predictive capability (Q²) of the OPLS-DA model were determined to be 0.969 and 0.787, respectively. Results indicate this model exhibits high classification accuracy and strong predictive capability for characteristic aroma components across different citrus varieties. The variable importance in projection (VIP) values from OPLS-DA can be used to evaluate each variable’s contribution to classification. Compounds with VIP > 1 were considered characteristic compounds. Higher VIP values indicate greater compositional differences among citrus varieties and a stronger contribution to sample differentiation. Results reveal a total of 115 characteristic aroma compounds with VIP values >1 (**Supplementary Table S2**). Among these, the differential metabolites safranal, cis−7−decen−1−al, borneol, endo−borneol, terpinen−4−ol, L-4-terpineol, and benzyl methyl sulfide exhibited particularly high VIP values (VIP > 2) (**Figure 4**). These compounds exhibit stronger aromas in ZYXC, XC, and CA, moderate aromas in YM, TN, KT, ZR, L131, and XY, and weaker aromas in KPS and SCM. In addition, 4−heptanol, 3−methyl−, citral, bis(2−chloroethyl) ether are characteristic aroma compounds of KBS and SCM (**Figure 4**; **Supplementary Figure S2**). α−farnesene, undecanol−5, and 1−undecanol were more abundant in ZYXC. Camphene, benzene, tertbutyl−, L-camphene, β-thujene, 1-methylformylpyrrole, propanoic acid, pentyl ester, hexanoic acid, ethyl ester, and butanoic acid, 1−methylbutyl ester accumulated more significantly in the CA sample (**Figure 4**; **Supplementary Figure S2**). These characteristic VOCs possess numerous flavors, resulting in the diverse aromatic profiles exhibited by different citrus fruits. For instance, the top 30 characteristic VOCs account for the variations in scents such as fresh, herbal, citrus, woody, camphor, fruity, sweet, and green among these citrus varieties (**Supplementary Figure S3**).

### 2.6 Gene co-expression network for VOCs

Key VOCs in citrus possess high rOAV values and play a major role in the formation of citrus aroma. Characteristic VOCs contribute to the aromatic differences among various citrus varieties. To elucidate the molecular regulatory mechanisms underlying citrus aroma formation and varietal divergence, we selected three VOCs with the strongest olfactory impact (highest rOAVs) and one VOC showing the greatest variation among cultivars (highest VIP value) for further analysis. Among the VOCs with the strongest aroma intensity, 1-p-menthene-8-thioli, also known as grapefruit mercaptan, is the primary aroma compound found in grapefruit. 3-mercapto-3-methylbutyl formate is a carboxylic acid ester widely used as a flavoring agent, characterized by intense catty, caramel, onion, roasted coffee, and sulfurous notes. 3-octen-2-one is a ketone with a potent earthy and mushroom-like odor, commonly applied in flavor and fragrance formulations. Among the VOCs exhibiting the greatest aromatic variation across citrus varieties, safranal is a monoterpene compound presenting as a pale straw-colored oily liquid with fresh, herbal, and rosemary-like aromas. To elucidate the gene regulatory mechanisms underlying the synthesis pathways of these important VOCs, transcriptome analysis was conducted. Weighted Gene Co-expression Network Analysis (WGCNA) was employed as a systems biology approach to identify modules of highly correlated genes across large-scale gene expression datasets. This method constructs a weighted gene co-expression network, clusters genes into distinct modules, and relates module eigengenes to VOC abundance, thereby enabling the identification of gene networks associated with specific VOC. Moreover, WGCNA facilitates the detection of hub genes within each module, which may serve as core regulators of VOC biosynthesis. To construct the gene co-expression network based on the appropriate scale-free topology criterion, set the soft-thresholding power of β = 14, MinModuleSize = 25, and MergeCutHeight = 0.75 (**Supplementary Figure S4**). Correlation analysis between module eigengenes and VOC levels revealed that the green module exhibited a strong positive correlation with 1-p-menthene-8-thiol (r = 0.85). The tan module showed high correlations with both 3-mercapto-3-methylbutyl formate (r = 0.90) and 3-octen-2-one (r = 0.89), while the darkgreen module was correlated with safranal (r = 0.70) (**Supplementary Figure S5**). These results indicate that these gene co-expression modules are closely involved in regulating the biosynthesis of their corresponding VOCs.

### 2.7 Hub gene for key VOCs

**Figure 5:**
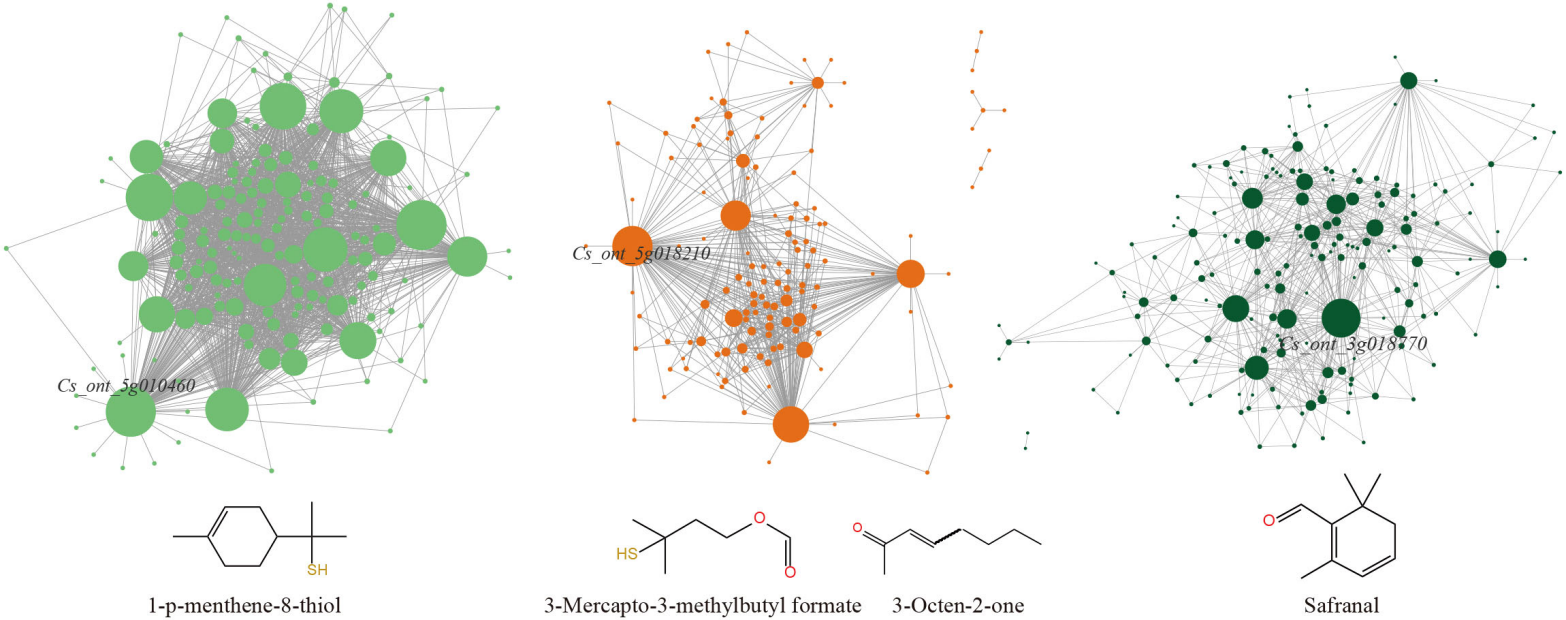
Visualization of key VOCs-related co-expression networks. The green module (left panel), tan module (middle panel), and darkgreen module (right panel) respectively show the interconnected relationships among gene nodes within each module, along with their corresponding VOCs displayed below the modules. Within the interactive network, node size is proportional to node degree. Selected key hub gene names are labeled.

**Figure 6:**
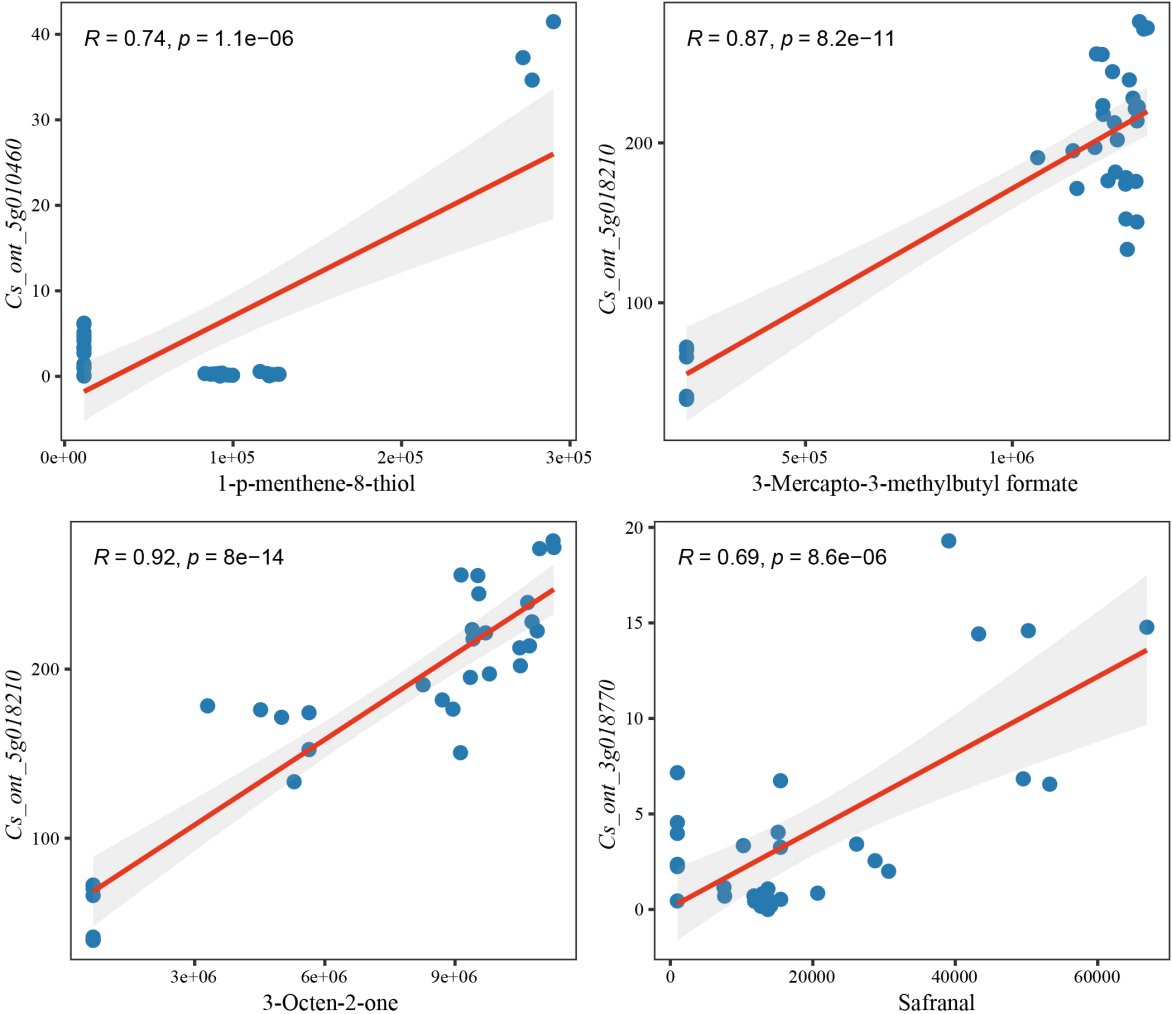
Scatter plot of key VOCs and their associated hub genes. The vertical axis represents gene expression levels across different citrus varieties, while the horizontal axis shows the relative content of VOC.

Genes with the highest degree of connectivity in essential modules are known as hub genes, and they may have important biological functions related to traits. The co-expression network was visualized using Cytoscape, and genes with the highest connectivity within each key module were identified as hub genes (**Supplementary Table S4**). As shown in the **Figure 5**, *Cs_ont_5g010460* was identified as the hub gene in the green module and was closely associated with the regulation of 1-p-menthene-8-thiol biosynthesis. The tan module’s hub gene, *Cs_ont_5g018210*, is highly associated with the synthesis of 3-Mercapto-3-methylbutyl formate and 3-Octen-2-one. The darkgreen module’s hub gene, *Cs_ont_3g018770*, plays a crucial role in safranal synthesis. To further demonstrate the close relationship between hub genes and their corresponding key VOCs, Pearson correlation analysis was conducted. The results showed a significant positive correlation between the expression level of *Cs_ont_5g010460* and the accumulation of 1-p-menthene-8-thiol (r = 0.736). Similarly, *Cs_ont_5g018210* exhibited strong positive correlations with 3-mercapto-3-methylbutyl formate (r = 0.865) and 3-octen-2-one (r = 0.916). In addition, *Cs_ont_3g018770* showed a significant correlation with safranal content (r = 0.691) (**Figure 6**). These results indicate that the expression levels of these hub genes are closely associated with the accumulation patterns of their respective VOCs, supporting their potential regulatory roles in citrus aroma formation.

**Figure 7:**
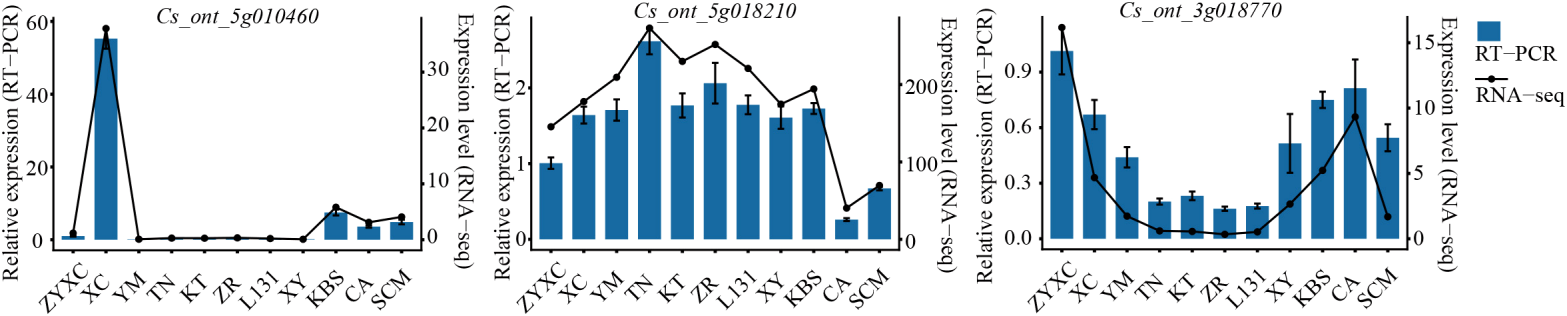
Hub gene expression levels were determined by RT-PCR and RNA-seq, respectively.

To further validate the expression patterns of the hub genes, RT-PCR analysis was conducted. The results showed that the expression levels of *Cs_ont_5g010460*, *Cs_ont_5g018210*, and *Cs_ont_3g018770* determined by RT-PCR were highly consistent with the RNA-seq data, confirming the reliability of the transcriptome analysis for quantifying gene expression (**Figure 7**). *Cs_ont_5g010460* exhibited significantly higher expression in XC than in the other citrus varieties, which was consistent with the elevated accumulation of 1-p-menthene-8-thiol in XC. *Cs_ont_5g018210* showed relatively low expression levels in CA and SCM, corresponding to the reduced accumulation of the key VOCs 3-mercapto-3-methylbutyl formate and 3-octen-2-one in these varieties. *Cs_ont_3g018770* was highly expressed in ZYXC, XC, and CA, displaying an expression pattern similar to the accumulation trend of the characteristic VOC safranal (**Supplementary Figures S2**, **S3**, **S7**).

Functional annotation further revealed the putative biological roles of these hub genes. *Cs_ont_5g010460* encodes ovate family protein 16 (OFP16), a plant-specific transcriptional regulator that functions as a transcription factor or repressor and plays an important role in modulating downstream gene expression. *Cs_ont_5g018210*, encoding SKP1-interacting partner 16 (SKIP16), is an F-box protein and a core component of the SCF (SKP1–Cullin–F-box) ubiquitin ligase complex. This protein mediates the selective recognition and ubiquitination of target substrates, thereby regulating protein turnover through the 26S proteasome pathway. *Cs_ont_3g018770* encodes a zeta-carotene desaturase-like (ZDS-like) protein, whose homologs catalyze the introduction of double bonds into colorless zeta-carotene, leading to the formation of colored carotenoids such as lycopene (Chen et al., 2017). This enzyme is essential for the biosynthesis of metabolites, plant development, and the enhancement of crop nutritional quality. Based on their potential biological functions, these genes may control the biosynthesis of these key VOCs by regulating gene transcription, protein levels, and catalytic reactions.

## 3 Discussion

Aroma is a defining quality trait of citrus fruits and plays a particularly important role in the utilization and commercial value of aromatic acidic citrus germplasm. Despite the economic and ecological significance of aromatic acidic citrus, systematic studies integrating aroma profiling with molecular regulatory mechanisms remain limited. In this study, we combined electronic nose analysis, volatile metabolomics, and transcriptome-based co-expression network analysis to comprehensively characterize aroma composition and its genetic regulation across representative aromatic acidic citrus varieties. Our results provide new insights into the diversity of citrus aroma profiles and the transcriptional networks underlying key aroma-related metabolites.

E-Nose analysis revealed that most aromatic acidic citrus varieties shared broadly similar aroma response patterns, whereas Kabosu and citrumelo displayed clearly distinct olfactory profiles (**Figure 1B**). This observation was consistently supported by multivariate analyses of both E-nose data and VOC metabolomic profiles, indicating that the divergence in aroma perception is driven by underlying chemical composition rather than sensor artifacts (**Figures 1C, D**, **2**). However, electronic nose responses do not always show a perfect correspondence with VOC compositional profiles. Notably, although the VOC composition of the citrange variety was more similar to that of Kabosu and citrumelo, its E-nose response pattern was closer to that of other citrus cultivars (**Figures 1C, D**, **2**). This discrepancy may be attributed to differences in the relative abundance of high-impact odorants with low odor thresholds, as well as to the selective sensitivity of metal oxide sensors toward specific functional groups. Electronic noses primarily capture integrated olfactory responses weighted by sensor sensitivity rather than the full qualitative and quantitative spectrum of VOCs, which can result in perceptual similarities despite compositional differences. In addition, synergistic or masking effects among VOCs may further influence sensor responses, leading to partial decoupling between metabolomic profiles and E-nose signals.

The strong contributions of sensors W5S, W1S, W1W, W2S, and W2W suggest that sulfur-containing compounds, terpenoids, alcohols, aldehydes, and ketones collectively dominate citrus aroma perception (**Figure 1B**) (Zhai et al., 2023). These findings are consistent with previous reports showing that citrus aroma is shaped by a limited subset of VOC classes with strong odor activity rather than by total VOC abundance alone (Yang et al., 2021; Wang et al., 2022). Phylogenetic and phenotypic analyses further demonstrated that genetic relatedness strongly influences aroma similarity, particularly among yuzu cultivars. Japanese and Korean yuzu varieties exhibited both closer evolutionary relationships and more homogeneous VOC profiles compared with Chinese yuzu cultivars (**Figures 1A**, **2D**). This pattern suggests that domestication history and regional selection may have contributed to the stabilization of aroma traits within local yuzu populations.

Volatile metabolomic analysis identified terpenoids as the most abundant and diverse class of VOCs across all aromatic acidic citrus varieties, in agreement with previous studies on citrus essential oils (González-Mas et al., 2019). However, rOAV-based evaluation revealed that compounds with exceptionally low odor thresholds, particularly sulfur-containing VOCs, exert a disproportionate influence on aroma perception. Among these, 1-p-menthene-8-thiol, 3-mercapto-3-methylbutyl formate, and 3-octen-2-one consistently exhibited the highest rOAV values and ranked among the dominant contributors to citrus aroma across varieties. These compounds are known to impart characteristic citrus, sulfurous, and mushroom-like notes, which are critical for the freshness and recognizability of citrus aroma. Notably, these high-impact VOCs were more abundant in yuzu cultivars, providing a chemical explanation for the intense and distinctive fragrance of yuzu widely appreciated in culinary and industrial applications (**Figure 3**). This finding underscores the importance of considering odor activity rather than absolute concentration. Beyond universally important aroma compounds, OPLS-DA analysis identified a set of characteristic VOCs with high VIP values that distinguish different aromatic acidic citrus varieties (**Supplementary Table S2**). Among these, safranal exhibited the greatest inter-varietal variation and emerged as a key discriminative compound. Safranal is a monoterpene-derived aldehyde with a fresh, herbal aroma and has been widely reported as a major aroma component in saffron and other aromatic plants (Esmaealzadeh et al., 2023). Its differential accumulation among citrus varieties suggests that specific branches of terpenoid metabolism contribute to varietal aroma identity. The accumulation patterns of other characteristic VOCs, such as borneol, terpinen-4-ol, and benzyl methyl sulfide, further illustrate the complexity of citrus aroma formation (**Figure 4**; **Supplementary Figure S2**). These compounds collectively generate diverse sensory notes, including citrus, woody, camphoraceous, sweet, and green aromas, explaining the marked sensory differences observed among aromatic acidic citrus varieties (**Supplementary Figure S2**). The presence of distinct VOC signatures in Kabosu, citrumelo, and citrange also supports their potential for targeted utilization in specialized processing applications (**Figure 2D**).

By integrating VOC profiling with transcriptome data, WGCNA enabled the identification of gene co-expression modules associated with key aroma compounds. The strong correlations between specific modules and high-impact VOCs indicate that aroma formation in citrus is regulated by coordinated transcriptional networks rather than by individual genes acting in isolation (**Supplementary Figure S5**). Importantly, different VOCs were associated with distinct co-expression modules, suggesting that multiple, partially independent regulatory pathways contribute to the overall aroma profile. Within these modules, several hub genes were identified as potential regulators of VOC biosynthesis. *Cs_ont_5g010460*, encoding an ovate family protein (OFP16), showed a strong association with 1-p-menthene-8-thiol accumulation (**Figure 6**). OFP family proteins are known transcriptional regulators involved in diverse developmental and metabolic processes, and their involvement in aroma regulation suggests a previously unrecognized role in volatile metabolism (Li et al., 2011; Wu et al., 2022; Zhang et al., 2023). *Cs_ont_5g018210*, encoding an F-box protein (SKIP16), was strongly correlated with 3-mercapto-3-methylbutyl formate and 3-octen-2-one, indicating that ubiquitin-mediated protein turnover may influence sulfur-containing and ketone-related VOC biosynthesis (**Figure 6**) (Ouni et al., 2011; Anand et al., 2012). *Cs_ont_3g018770*, encoding a zeta-carotene desaturase-like protein, was associated with safranal accumulation (**Figure 6**), Although ZDS is classically recognized as a core enzyme in carotenoid biosynthesis, its association with safranal suggests that enzymes involved in primary or secondary metabolic pathways may exert indirect regulatory effects on aroma compound formation. Such effects may arise through metabolic crosstalk, shared precursors, or the modulation of key intermediates and fluxes across interconnected pathways. Increasing evidence indicates that plant metabolic enzymes do not function exclusively within isolated pathways but instead participate in complex, interconnected metabolic networks. For example, phenylalanine ammonia-lyase (PAL), the first rate-limiting enzyme in phenylpropanoid biosynthesis, not only controls lignin accumulation but also influences the synthesis of flavonoids and salicylic acid, thereby coordinating multiple physiological processes (Reyes Jara et al., 2022; Zhu et al., 2025). This highlights the integrative nature of plant metabolic regulation, in which enzymes and regulatory proteins influence multiple branches of metabolism rather than acting as components of discrete metabolic factories. Although these associations do not establish direct causality, the strong correlations observed between gene expression and VOC accumulation provide compelling evidence that these hub genes represent key regulatory nodes in citrus aroma metabolism. Functional validation through gene editing or transgenic approaches will be necessary to confirm their precise roles. The integrated analysis presented here has important implications for both citrus processing and molecular breeding. From an applied perspective, the identification of key aroma compounds and characteristic VOC signatures provides a scientific basis for selecting aromatic acidic citrus varieties tailored to specific industrial uses. From a breeding standpoint, the hub genes identified in this study represent promising targets for aroma-oriented molecular breeding and metabolic engineering. Manipulating these regulatory genes may enable the enhancement or modification of aroma traits without adversely affecting other quality attributes.

Although this study provides an integrated analysis of aroma composition and its transcriptional regulation in aromatic acidic citrus, several limitations should be acknowledged. First, the present work primarily relied on correlation-based approaches, including rOAV evaluation and weighted gene co-expression network analysis, to infer the relationships between VOC accumulation and gene expression. While these analyses effectively identify candidate regulatory genes, direct functional validation through genetic or biochemical experiments is still required to establish causal relationships. Future studies employing gene editing, transgenic overexpression, or virus-induced gene silencing will be essential to confirm the regulatory roles of the identified hub genes in aroma biosynthesis. Second, although GC–MS is widely regarded as a robust and accurate method for VOCs detection and quantification, its performance can be influenced by compound-specific analytical challenges. In particular, structural isomers may lead to qualitative or quantitative ambiguities due to incomplete chromatographic separation or highly similar mass fragmentation patterns. Such issues are inherently related to molecular structure and mass spectrometric behavior and may result in identical or integer-multiple concentration estimates for certain compounds (e.g., Phenol, 2,4-dichloro-, Phenol, 2,5-dichloro-, and Phenol, 2,3-dichloro- in **Supplementary Figure S2**). Addressing these limitations will require further optimization of chromatographic conditions, careful selection of characteristic ions, or the adoption of advanced analytical strategies, such as comprehensive two-dimensional gas chromatography. Finally, although the electronic nose and GC–MS analyses captured a broad spectrum of VOCs, human sensory evaluation was not included. Combining instrumental analyses with trained sensory panels in future research would allow for a more direct linkage between chemical composition and perceived aroma quality. Such integrative approaches will be particularly valuable for translating molecular findings into practical strategies for citrus breeding and processing.

In summary, this study provides a comprehensive view of aroma diversity and its transcriptional regulation in aromatic acidic citrus. By linking sensory-related metabolites with gene co-expression networks, our findings advance the understanding of citrus aroma biosynthesis and offer valuable resources for the efficient utilization and genetic improvement of aromatic acidic citrus germplasm.

## 4 Materials and methods

### 4.1 Plant materials

This study employed the ‘Ziyangxiangcheng’ (ZYXC), ‘Xiecheng’(XC), ‘Kitou’ (KT), ‘Tada Nishiki’ (TN), ‘Zairai’(ZR), ‘Yamane’ (YM), ‘Line131’ (L131) yuzu (*Citrus junos*) cultivars. Additionally, ‘Kabosu’ (KBS) (*Citrus* sp*aerocarpa*), ‘Swingle’ citrumelo (SCM), Xiang yuan (XY) (*Citrus wilsonii*), and citrange (CA) were included. All citrus fruits were cultivated at the experimental field of Zhejiang Citrus Research Institute (E: 121.9, N: 28.38), with uniform cultivation practices applied throughout. By 1st December, all fruits had reached maturity with consistent ripeness levels. Citrus peel samples were randomly collected, immediately flash-frozen using liquid nitrogen, and stored at -80 °C for subsequent experiments. Three biological replicates were established for each cultivar.

### 4.2 Electronic nose analysis

The PNE3 electronic nose system (Airsense Analytics, Schwerin, Germany) was employed to analyse volatile compounds released from citrus peel samples. This olfactory system incorporates 10 different semi-conductive metal oxide semiconductor sensors responsive to distinct chemical classes, enabling comprehensive characterization of aroma profiles. For analysis, approximately 3g of citrus peel is placed within a sealed vial. The vial is then sealed and positioned within the sensor chamber of the PNE3 electronic nose system. The system operates at 25 °C with a carrier gas ambient air flow rate of 200 mL/min. Measurement parameters were based on the methodology described by Chen et al (Chen et al., 2020). Steady-state response values were recorded for 57 to 60 s during each test, with data analysis performed using WinMuster software (version 1.6.2).

### 4.3 Extraction of volatile organic compounds

VOCs were extracted using headspace solid-phase microextraction (HS-SPME). Approximately 0.5 g of ground sample was transferred into a 20 mL glass headspace vial (Agilent Technologies, Palo Alto, CA, USA) and sealed with a polytetrafluoroethylene (PTFE)/silicone septum. Sodium chloride was added to enhance VOC release by salting-out effects. The vial was equilibrated at 60 °C for 5 min with agitation. VOCs were extracted by exposing a 120 μm divinylbenzene/carboxen/polydimethylsiloxane SPME fiber to the headspace of the sample for 15 min at 60 °C. After extraction, the SPME fiber was immediately inserted into the injection port of the gas chromatograph for thermal desorption.

### 4.4 Metabolomic analysis of VOCs

VOC analysis was performed using a gas chromatography–mass spectrometry (GC–MS) system (Agilent 7890 GC coupled with a 5977 MSD). Separation was achieved on a DB-5MS capillary column (30 m × 0.25 mm × 0.25 μm, 5% phenyl-polymethylsiloxane) (Agilent Technologies, Palo Alto, CA, USA). The GC oven temperature program was set as follows: initial temperature at 40 °C for 3.5 min, increased to 100 °C at 10 °C min⁻¹, and finally to 280 °C at 25 °C/min (held for 5 min). Helium was used as the carrier gas at a constant flow rate of 1.2 mL min⁻¹. The MS was operated in electron impact (EI) mode at 70 eV, with a mass scan range of m/z 35–500. The ion source and quadrupole temperatures were set to 230 °C and 150 °C, respectively. VOCs were detected using selected ion monitoring (SIM) mode.

Relative quantification of VOCs was performed based on peak area normalization, and the results were expressed as the relative percentage of total ion chromatogram (TIC) peak areas. The normalized VOC data were subjected to multivariate statistical analyses, including principal component analysis (PCA) and Orthogonal Partial Least Squares Discriminant Analysis (OPLS-DA), using MetaboAnalyst package in R (version 4.5.1) (Pang et al., 2024). Metabolites with a variable importance in projection (VIP) value > 1.0 were considered as key VOCs.

### 4.5 RNA-sequencing and data analysis

Total RNA was extracted from citrus peels using the TRIzol reagent (Invitrogen, USA) following the manufacturer’s instructions. RNA concentration and purity were assessed using a NanoDrop 2000 spectrophotometer (Thermo Scientific, USA). RNA samples with an RNA integrity number (RIN) ≥ 7.0 were used for library construction. Each experimental group consisted of three independent biological replicates. Libraries were amplified by PCR and sequenced on a NovaSeq X Plus platform (Illumina, Inc., San Diego, CA, USA) to generate 150-bp paired-end reads by Metware Biotechnology Co., Ltd. (Wuhan, China). Raw sequencing reads were processed to remove adapter sequences, low-quality reads, and reads containing poly-N using fastp (version 0.23.4) (Chen et al., 2018). Clean reads were mapped to the *Citrus sinensis* reference genome using HISAT2 with default parameters (version 2.2.0) (Kim et al., 2015). Uniquely mapped reads were retained for calculating read counts. Gene expression levels were quantified as fragments per kilobase of transcript per million mapped reads (FPKM) using featureCounts (Liao et al., 2013).

Weighted gene co-expression network analysis (WGCNA) was performed using the WGCNA package in R to identify gene modules associated with key VOCs (Langfelder and Horvath, 2008). Genes with low expression levels (FPKM < 1) were filtered out prior to network construction to reduce noise. An expression matrix of the remaining genes was used to calculate pairwise Pearson correlation coefficients. A soft-thresholding power (β) was selected based on the scale-free topology criterion using the pickSoftThreshold function. The adjacency matrix was constructed and subsequently transformed into a topological overlap matrix (TOM), which measures the network connectivity between genes. Hierarchical clustering was performed based on the TOM dissimilarity measure, and gene modules were identified using the dynamic tree cut method with a minimum module size of 25 genes. Highly similar modules were merged using a threshold of 0.25. Each module was summarized by its module eigengene (ME), representing the first principal component of the gene expression matrix within a module. Module–trait relationships were evaluated by correlating ME with sample traits (VOC levels) using Pearson correlation analysis. Modules with high correlation coefficients and significant *p*-values were considered biologically relevant. Hub genes within key modules were identified based on highest degree of connectivity.

### 4.6 RT-qPCR analysis

Residual gDNA was removed and cDNA synthesized using the Hifair^®^ III 1st Strand cDNA Synthesis SuperMix for qPCR (YEASEN, Shanghai, China). 2×HS Taq Universal SYBR Green Qpcr Master Mix (SAIPUBIO, Shenzhen, China) and BIOER FQD-96A (BIOER, Hangzhou, China) were used for qPCR experiments. Actin3 was used as internal control gene in citrus. Three biological replicates were performed for each experiment and relative gene expression was calculated following the 2 ^-ΔΔCT^ method. Values are presented as mean values. Primer information is provided in **Supplementary Table S5**.

## Data Availability

The data that support the findings of this study are available from the corresponding author upon reasonable request. The RNA-seq data on which this article is based are available on the National Center for Biotechnology Information website at https://www.ncbi.nlm.nih.gov/ and can be accessed via PRJNA1399633.

## Supplementary material

Supplementary Figure 1 Flavor sankey diagram of the top 10 VOCs in various citrus varieties.

Supplementary Figure 2 Scatter plot of rOAV values for the top 10 VOCs in each citrus variety.

Supplementary Figure 3 Flavor sankey diagram of VOCs with higher VIP values across different citrus varieties.

Supplementary Figure 4 Network topology of different soft-thresholding power. The left graph shows the scale-free fit index corresponding to various soft-thresholding power (β). The panel on the right shows the mean connectivity corresponding to various soft-thresholding power (β)

Supplementary Figure 5 Heatmap showing the correlation between gene co-expression modules and specific key VOCs.
