# Supplementary figures and images for: Integrated metabolomic and transcriptomic analyses reveal aroma diversity and its regulatory networks in aromatic acidic citrus
Source: Front Plant Sci. 2026 Feb 20;17:1785725. doi: 10.3389/fpls.2026.1785725 (PMC12963248; doi:10.3389/fpls.2026.1785725)

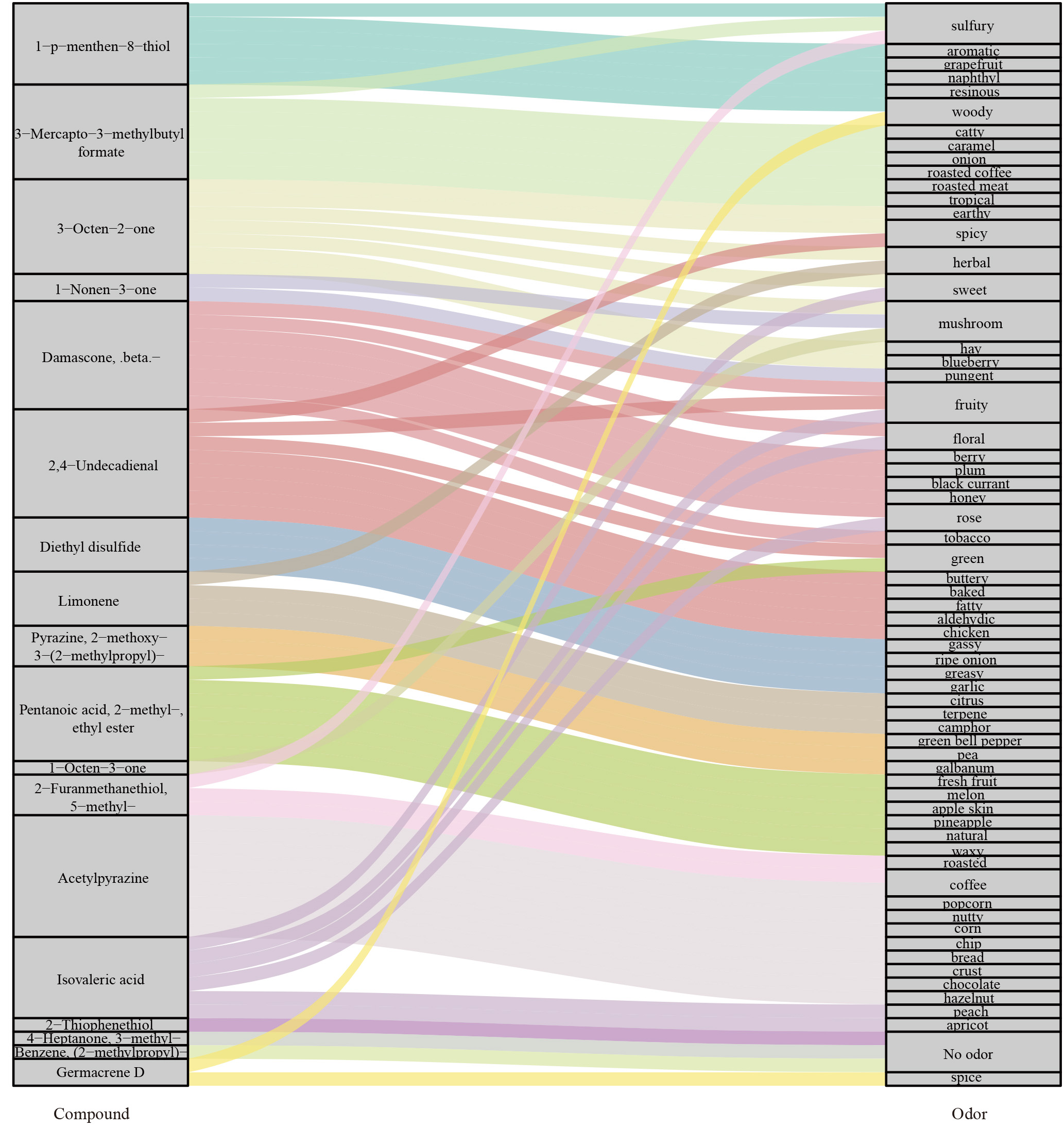

Supplement: Supplementary Figure 1 — Flavor sankey diagram of the top 10 VOCs in various citrus varieties. [file Image1.jpeg]

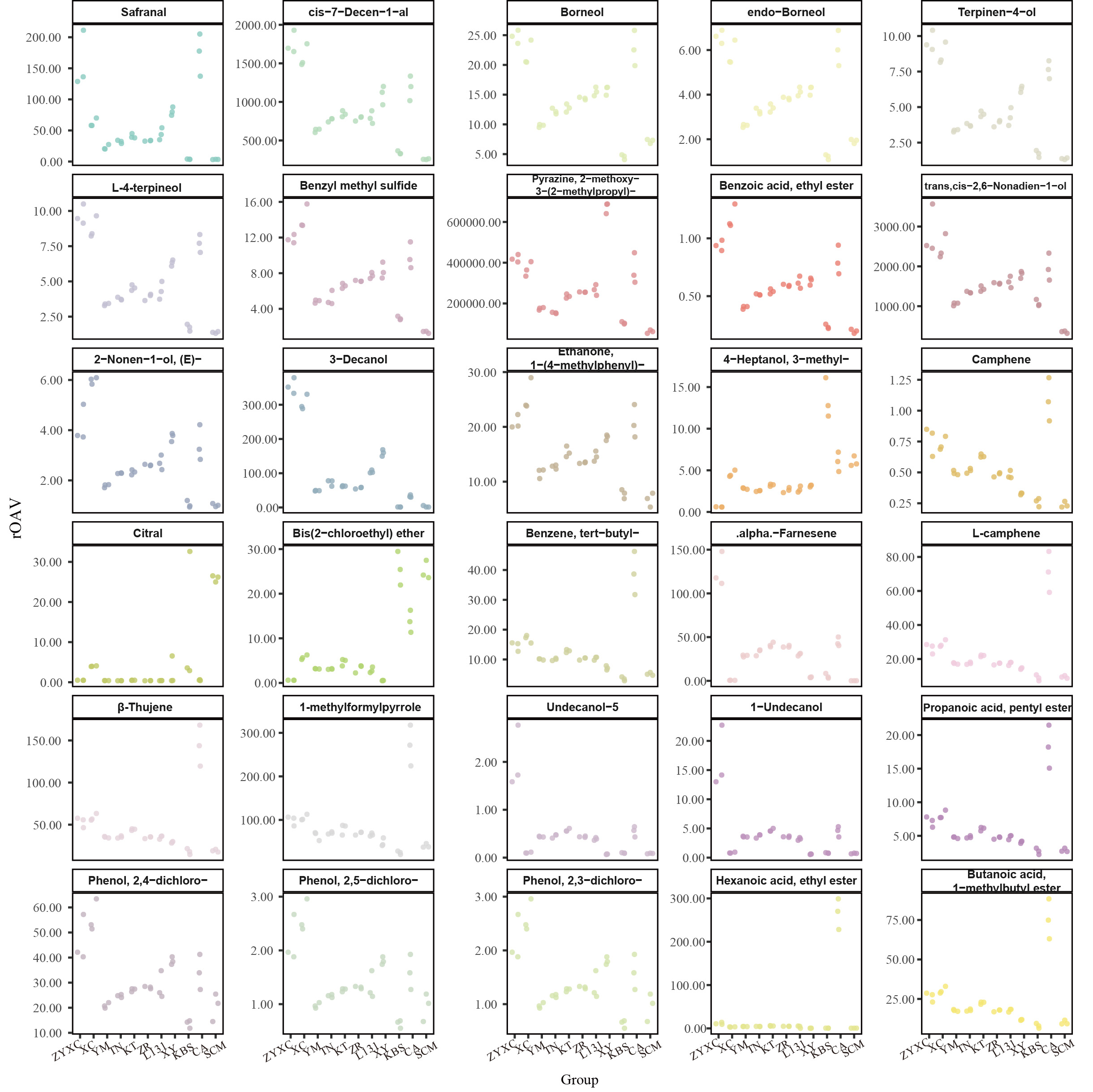

Supplement: Supplementary Figure 2 — Scatter plot of rOAV values for the top 10 VOCs in each citrus variety. [file Image2.jpeg]

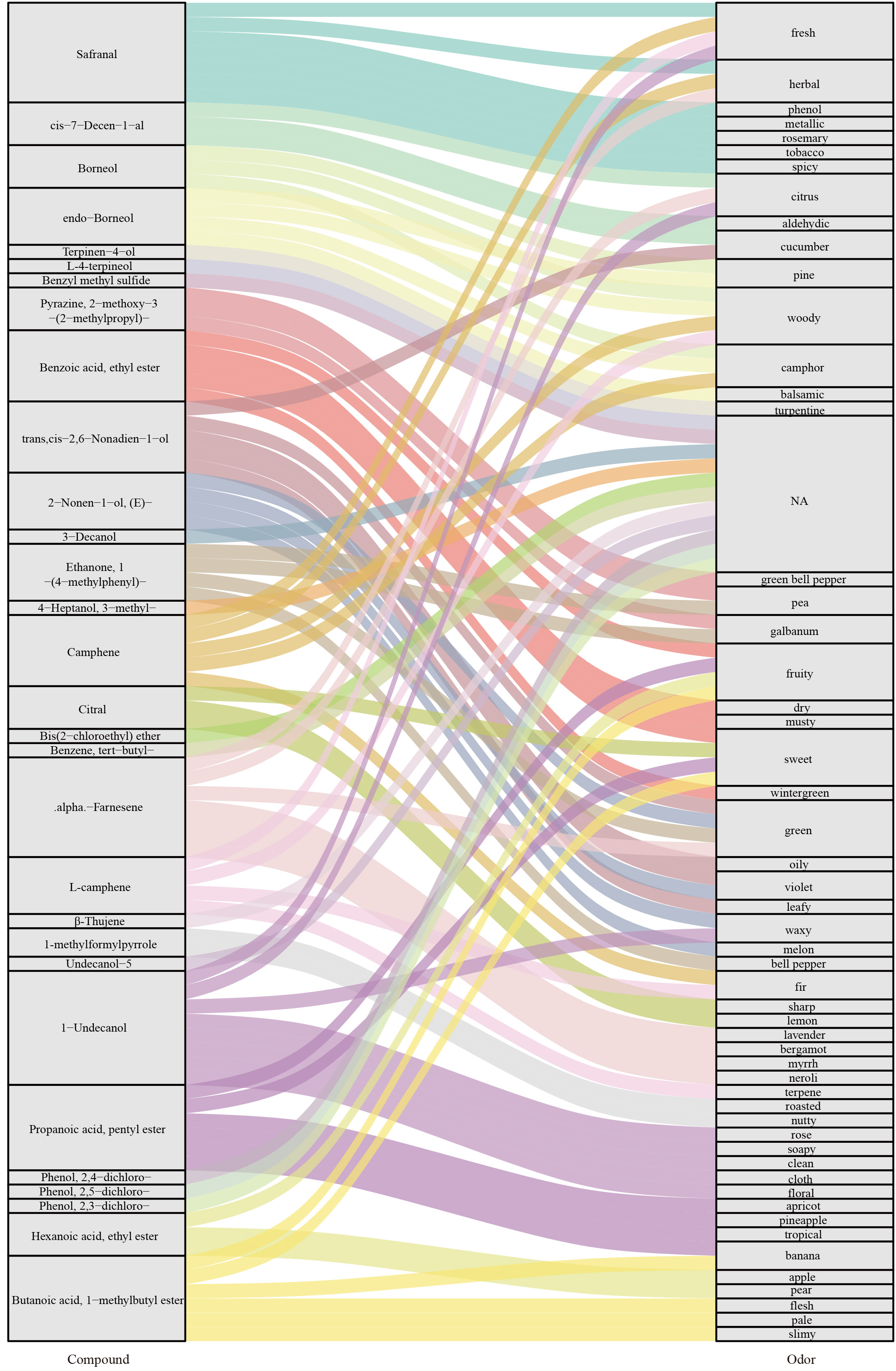

Supplement: Supplementary Figure 3 — Flavor sankey diagram of VOCs with higher VIP values across different citrus varieties. [file Image3.jpeg]

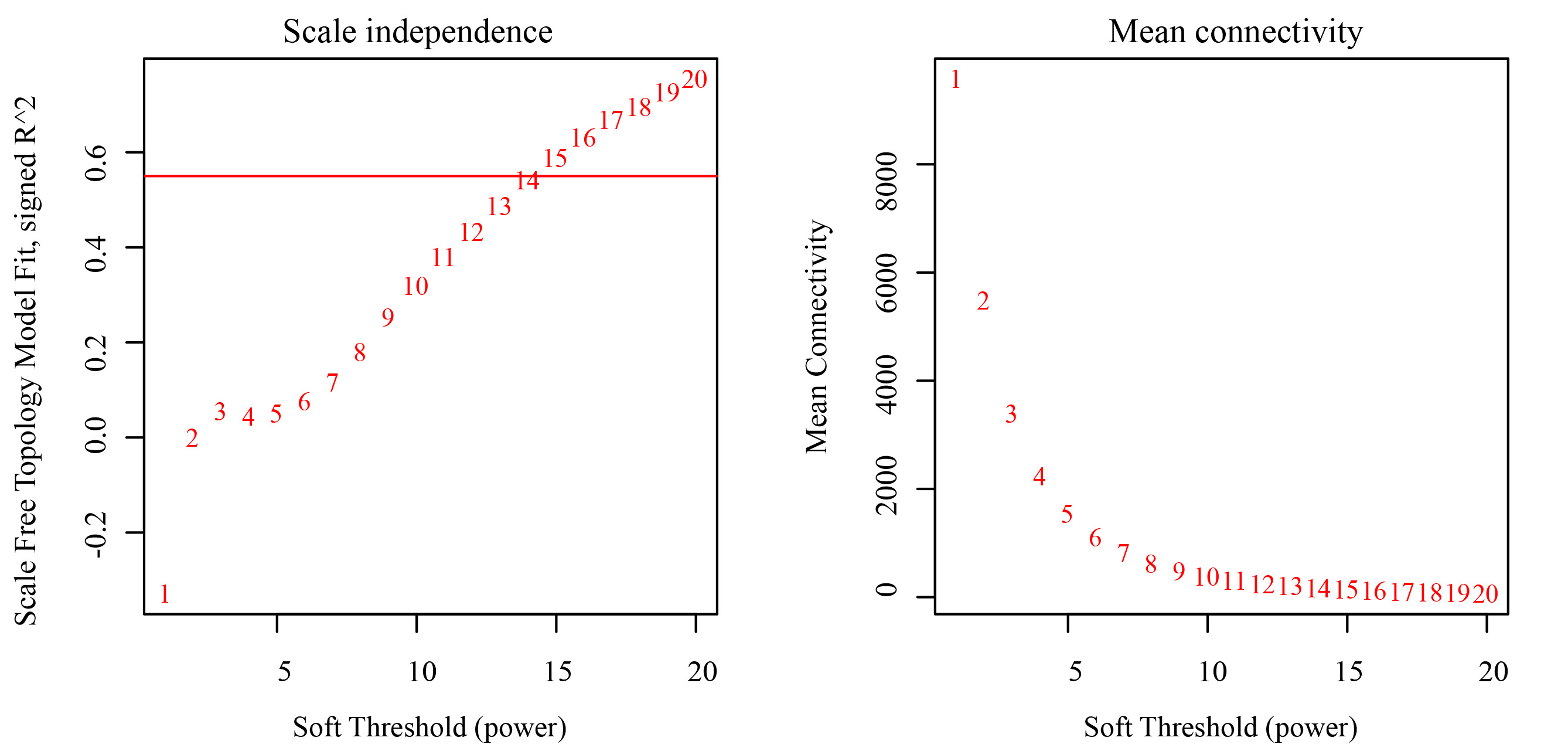

Supplement: Supplementary Figure 4 — Network topology of different soft-thresholding power. The left graph shows the scale-free fit index corresponding to various soft-thresholding power (β). The panel on the right shows the mean connectivity corresponding to various soft-thresholding power (β) [file Image4.jpeg]

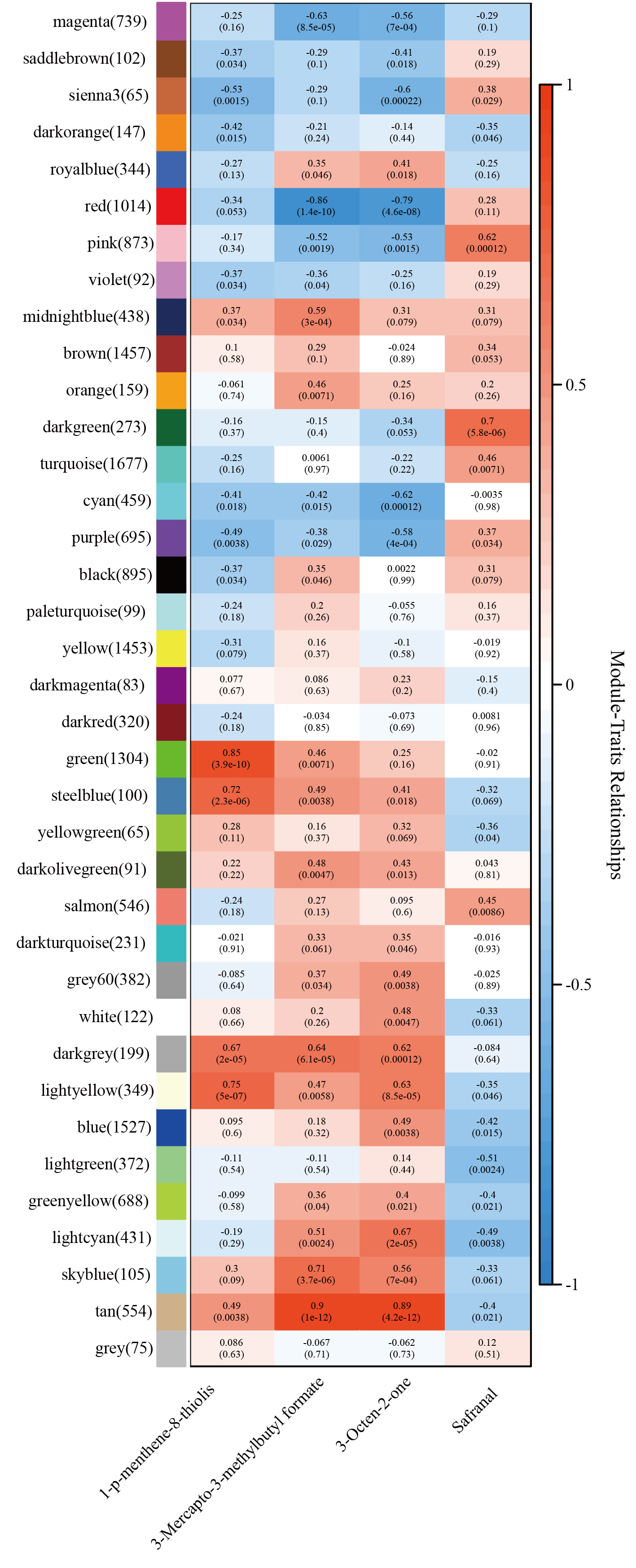

Supplement: Supplementary Figure 5 — Heatmap showing the correlation between gene co-expression modules and specific key VOCs. [file Image5.jpeg]
